# Classic vs. Novel Antibacterial Approaches for Eradicating Dental Biofilm as Adjunct to Periodontal Debridement: An Evidence-Based Overview

**DOI:** 10.3390/antibiotics11010009

**Published:** 2021-12-22

**Authors:** Ali Abdulkareem, Hayder Abdulbaqi, Sarhang Gul, Mike Milward, Nibras Chasib, Raghad Alhashimi

**Affiliations:** 1 College of Dentistry, University of Baghdad, Medical City of Baghdad, Baghdad 10011, Iraq; raad.hayder@gmail.com (H.A.); dr.nbras@gmail.com (N.C.); raghad.alhashimi@gmail.com (R.A.); 2 College of Dentistry, University of Sulaimani, Sulaymaniyah 40062, Iraq; sarhang.hama@univsul.edu.iq; 3 College of Dentistry, University of Birmingham, Birmingham B5 7EG, UK; m.r.milward@bham.ac.uk

**Keywords:** antibacterial, biofilms, periodontal debridement, bacterial resistance

## Abstract

Periodontitis is a multifactorial chronic inflammatory disease that affects tooth-supporting soft/hard tissues of the dentition. The dental plaque biofilm is considered as a primary etiological factor in susceptible patients; however, other factors contribute to progression, such as diabetes and smoking. Current management utilizes mechanical biofilm removal as the gold standard of treatment. Antibacterial agents might be indicated in certain conditions as an adjunct to this mechanical approach. However, in view of the growing concern about bacterial resistance, alternative approaches have been investigated. Currently, a range of antimicrobial agents and protocols have been used in clinical management, but these remain largely non-validated. This review aimed to evaluate the efficacy of adjunctive antibiotic use in periodontal management and to compare them to recently suggested alternatives. Evidence from in vitro, observational and clinical trial studies suggests efficacy in the use of adjunctive antimicrobials in patients with grade C periodontitis of young age or where the associated risk factors are inconsistent with the amount of bone loss present. Meanwhile, alternative approaches such as photodynamic therapy, bacteriophage therapy and probiotics showed limited supportive evidence, and more studies are warranted to validate their efficiency.

## 1. Introduction

Since their discovery, the use of antibiotics has not been limited to treating diseases affecting humans, and their extended application to veterinary medicine and agriculture has been directly implicated in the development of bacterial resistance [1,2]. Additionally, the presence of antibiotics in wastewater from antibiotic manufacturers and hospitals is greatly contributing to large-scale environmental pollution and the development of bacterial resistance [3]. Unfortunately, the threat from bacterial resistance is a very pressing issue that has major ramifications to public health and economies. For instance, in the USA, the cost of hospitalization that is directly due to antimicrobial resistance is over $35 billion per year [4]. In addition, some of the older well-established antibiotics cannot counteract highly resistant-bacterial species including *Streptococcus pneumoniae, Streptococcus pyogenes*, and *Staphylococcus aureus,* which are responsible for respiratory and cutaneous infections, and a considerable number of mortalities each year [5]. To compound the problem, since the 1980s, the introduction of new antimicrobials has decreased steadily [6,7]. Since then, the available antibiotics have been either modifications or combinations of classic antibiotics (about 73% of new antibiotics are based on older antibiotics such as penicillin and quinolones) [8] which potentially will be overwhelmed by the rapid increase in bacterial resistance. Consequently, this is a worsening global crisis with the potential to overburden health care systems and economies in the coming decades [9].

The use of antibiotics for managing oral disease is a popular practice amongst dentists; however, it is important they are only used in specific situations such as in curtailing the spread of infection or in preventing systemic involvement. Antibiotics may also be advocated where a bacterial procedure may lead to the risk of endocarditis, although opinions of the need vary [10,11,12]. The availability of antibiotics in some countries can lead to overuse, and this is cited as a major cause for developing antimicrobial resistance [13]. Consistently, the misuse of antibiotics by dentists has been reported as a significant issue [14].

Periodontitis is an inflammatory disease initiated by dysbiosis of the subgingival microbiome, with aberrant immune response, causing collateral damage to the tooth-supporting tissues and ultimately leading to tooth loss [15,16]. Dental plaque biofilm is considered as the primary etiologic factor for the majority of dental/periodontal diseases [17]. The gold standard of treatment for periodontitis is mechanical debridement of subgingival biofilm. Indeed, suppression of pathogenic microorganisms has for a long time been a keystone in regeneration and repair of periodontal tissues, which can be challenging using mechanical debridement alone, which must be complemented by patient-based plaque control programs [18,19,20]. For many decades, attempts have been made to improve the efficacy of mechanical treatment by introducing different adjuncts such as the use of antimicrobials/antibiotics at different dosages and routes of administration. However, the structural complexity of dental biofilm provides a shelter for many pathogenic microorganisms, making delivery to individual bacteria challenging [21]. In addition, due to the non-specificity of these drugs, they may target useful commensal species which counteract pathogenic biofilm development [22].

The aforementioned challenges and limitations for using antibiotics during periodontal treatment have motivated researchers to find alternative and more efficient adjunctive approaches. Therefore, the aim of this review was to summarize the available literature, determining efficacy of using antibiotics during periodontal therapy and the effectiveness of alternative methods.

### Search Strategy

The literature were retrieved from three search engines: PubMed (National Library of Medicine), Cochrane Library (Wiley), and Medline (EBSCO) using the following search terms:

(antibiotics) OR (antimicrobials) AND (nonsurgical periodontal therapy) OR (periodontal therapy) AND (periodontal bacteria) AND (antimicrobial resistance) OR (bacterial resistance) AND (anti-infective agents) AND (periodontitis) OR (Periodontal disease).

Studies were selected without quality assessment. However, certain inclusion criteria were followed for selecting randomized clinical trials (RCTs) and controlled clinical trials:

- Follow-up for at least three months
- One of the arms received subgingival debridement (SD) with adjunctive antimicrobial or photodynamic therapy or probiotics. The other arm (control) should receive SD alone.
- Reporting both microbiological and clinical outcomes

For observational and in vitro studies, selection was mainly based on studies targeting orange and/or red complex periodontal bacteria. Articles were excluded if they were not written in English or were not original studies e.g., reviews, short communications, case reports/series.

**Figure 1:**
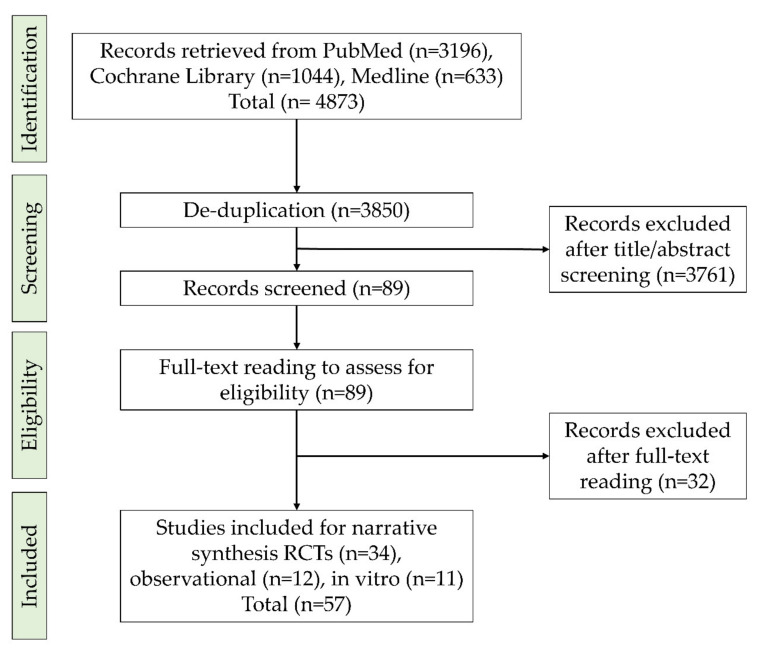
Flowchart of the study selection.

Retrieved articles were screened for duplicates and then the remaining articles were assessed and discussed for their eligibility by three reviewers (A.A., H.A., and S.G.) starting with title, abstract, and finally full-text reading. Results of the search procedure are summarized in Figure 1.

## 2. Structure of Biofilm

Dental plaque biofilm is formed on oral surfaces and composed of microorganisms embedded within an intercellular matrix [23]. The formation of dental biofilm is a complex process that passes through several sequential steps. Briefly, the attachment of salivary glycoproteins on clean tooth surfaces to form the acquired pellicles is the initial step. Several planktonic bacteria in saliva, such as *Actinomyces* spp. and *Streptococcus* spp., attach to binding proteins on the surface of acquired pellicles. These pioneer bacteria utilize their appendages such as fimbria and fibrils to enhance their firm adherence to the acquired pellicles and start to excrete extracellular polymeric substances (EPS) to act as a ground substance for the biofilm. Moreover, they provide specific binding sites for the adhesion of the subsequent bacterial colonization. After the sequential competitive adhesion and colonization of bacteria, the dental biofilm expands and matures within days [24].

The microorganisms in dental biofilms are mainly bacterial cells, with over 600 species of bacteria having been identified within the biofilm [23]. These bacteria are arranged in microcolonies forming about 15% to 20% of the biofilm volume. The organization of the microcolonies is not even within the layers of the biofilm. The microorganisms are well-organized in deep layers forming a dense layer of microbes, while the superficial layers contain loosely organized microbes. Consequently, dental biofilms appear as an irregular mass and may blend with the surrounding medium [25]. Each microcolony contains multiple species of bacteria. The proximity of bacterial cells allows gene exchange and quorum sensing between the cells [24]. The latter is a communication mechanism that is induced with increasing density of bacteria in the biofilm resulting in regulation of gene expression, thereby controlling certain biological processes such as symbiosis, virulence, stress adaptation, and biofilm formation [26].

The backbone of the biofilm is the extracellular matrix where the microorganisms are embedded. This matrix is porous and contains water channels that act as routes for supplying bacteria with nutrients and disposing of waste products [24]. The matrix is composed of inorganic as well as organic materials. These are mainly derived from gingival crevicular fluid, saliva and bacterial products such as EPS [25]. EPS act as selective barriers surrounding biofilms that trap nutrients from the outside environment and shelter the bacteria from it. Additionally, EPS can repel harmful agents and protect resident bacteria from outside attack [24]. Furthermore, EPS can bind to antimicrobial substances and limit the diffusion of these substances within biofilms, thus protecting biofilm bacteria [27]. For that reason, most bacteria which survive in dental biofilms have the advantage of being more resistant to antibiotic agents from planktonic peers. However, their resistance is increased when the biofilms become more mature with increased varieties of bacterial species [28]. Therefore, prescription of local or systemic antibiotics/antimicrobials is not recommended as monotherapy for periodontal disease, as their action is minimized or neutralized unless the biofilm is mechanically disrupted [21].

## 3. Management of Dental Biofilm

### 3.1. Periodontal Debridement: The Gold Standard for Periodontal Therapy

The objective of periodontal therapy is removal of the causative factor, i.e., dental biofilm from tooth surfaces. In the majority of cases, patients’ oral hygiene measures are adequate to resolve gingivitis. This may be accomplished by mechanical debridement to remove hard deposits from teeth which enhance retention of dental biofilm [29]. In periodontal pockets, SD is pivotal for the removal of hard and soft sub-gingival deposits. To date, SD is the most effective method in the treatment of periodontitis [29]. It aims to remove the bulk of the dental biofilm, together with calculus which acts as a plaque-retentive factor and absorb bacterial toxins, thereby lowering the periodontal pathogens levels at subgingival sites, hence promoting recovery of periodontal health [29] by maintaining the level of periodontal pathogens to a threshold compatible with periodontal health [30]. This can be seen clinically through the reduction of inflammation and probing pockets depth (PPD), and the gain in clinical attachment levels (CAL) after SD using either hand or machine-driven instruments [31]. Therefore, periodontal debridement, with or without adjuncts, is still the gold standard modality for the treatment of periodontal diseases. However, long-term success is only ensured when the patients practice oral hygiene measures regularly [32].

### 3.2. Adjunctive Systemic and Local Antimicrobials/Antibiotics in Periodontics

**Figure 2:**
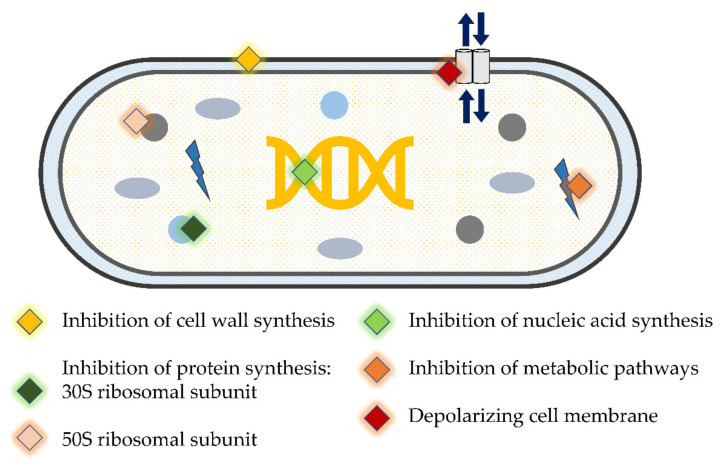
Mode of action of antimicrobial agents.

Although periodontitis is not related to specific bacteria, a number of periodontal pathogens have been identified. One of the goals of periodontal therapy is to move from a ‘pathogenic’ to a ‘healthy’ biofilm [33]. SD does not always produce the desired clinical improvement in all subjects or for the same subject in the long term [34,35]. This could be attributed to the operator’s lack of skill, the presence of inaccessible areas such as multi-rooted teeth, and/or the colonization by tissue invading species such as *Porphyromonas gingivalis* and *Aggregatibacter actinomycetemcomitans* that cannot be completely eradicated by SD alone [36,37]. Thus, other forms of treatment modalities such as antibiotics/antimicrobials have been proposed as adjunctive therapy [38,39]. These therapeutic agents have diverse mechanisms of action (Figure 2); either by inhibiting cell wall synthesis, acting on cell membrane, inhibiting RNA/DNA synthesis, interfering with metabolic pathways, and inhibiting protein synthesis [40]. However, according to the 6th European Workshop consensus statement, the administration of antibiotics has to be limited to certain patients, i.e., periodontitis grade C in young adults previously known as “aggressive periodontitis” [41]. Evidence from previous studies has demonstrated the effectiveness of adjunctive antibiotic administration in severe and progressing forms of periodontitis, mainly in the reduction of PPD, bleeding on probing (BOP) and CAL gain in comparison with SD alone [42,43,44]. However, it is important to highlight those sites with initially shallow to moderate PPD (4–5 mm) that do not show statistically significant improvement in clinical parameters (reduction in PPD and gain of CAL) after the adjunctive use of systemic antibiotics [45,46]. The prescription of antibiotics according to the latest classification of periodontal disease, including the exact stage and grade of periodontitis, remains to be determined. However, in a recent meta-analysis, statistically significant improvement in the clinical outcomes has been reported following adjunctive antimicrobial administration alongside periodontal therapy in subjects with periodontitis stages III/IV, and grade C, with no other risk factors [47].

If suitable regimes can be identified where appropriate use of adjunctive antibiotics has shown a clinical benefit, this could result in a decreased need for repeated non-surgical and/or surgical periodontal interventions [48,49,50]. This has many advantages for the patient, such as reducing hard tissue trauma to non-responsive sites as well as avoiding the high emotional and financial costs of surgical intervention [51]. Furthermore, smoker subjects with deep PPD might specifically benefit from adjunctive antibiotic prescription in the non-surgical phase; however, the correct action is smoking cessation for such patients [52].

#### Protocols of Antimicrobials/Antibiotics Prescription during Periodontal Therapy

Globally, dental practitioners frequently ask about when, what type, and for how long antibiotics should be prescribed for patients with periodontal disease. Unfortunately, pattern and dosage of antibiotic prescription are not consistent among dentists who usually find themselves in a grey zone due to lack of international standardized guidelines for using antibiotics in their practice [53]. Interestingly, the only clear guidelines are those issued by the American Heart Association on using prophylactic antibiotics with dental procedures to minimize the risk of cardiac complications [12].

According to the latest guidelines for treating periodontitis (stage I to III), prescribing antibiotics for periodontitis patients is not recommended. The only exception is rapidly progressing periodontitis in young adults in which amoxicillin (AMX) and metronidazole (MET) can be used either alone or as a combination [29]. Using AMX + MET simultaneously with SD results in statistically significant pocket closure, CAL gain, and BOP as compared to SD alone [47]. However, the long-term efficacy (>12 months) of this regimen on periodontal status has not yet been assessed.

Administration of systemic MET + AMX as an adjunct to SD in patients with aggressive periodontitis (currently periodontitis grade C in young adults) resulted in greater PPD reduction and CAL gain as compared to SD alone [54]. In addition, using this combination of antibiotics also enhanced the pocket closure when combined with SD [43]. These results were supported by a recent study which demonstrated the best clinical outcomes were obtained when MET + AMX were used with SD [55]. However, the aforementioned studies did not specify the optimal dose/duration for these antibiotics due to heterogeneity of doses and durations. The answer regarding this point can be concluded from results of a systematic-review and meta-analysis which recommended administrating a combination of AMX and MET at the highest dose for the shortest period (500/400 mg for 7 days), thereby reducing the risk of developing bacterial resistance [56]. Combination of AMX + MET also seems to be more beneficial for diabetic patients with periodontitis than other protocols [57]. Nevertheless, with no specific recommendations to follow for diabetics or smokers, the use of systemic antibiotics should be in the context of full-mouth debridement in healthy and nonsmoker individuals [29]. Based on evidence from in vitro studies, the tolerance of biofilm to antibiotics peaks within 24 h; therefore, it is advisable to start taking systemic antibiotics within hours after full-mouth debridement.

Although combining antibiotics with SD could yield better reduction in PPD and CAL gain, their prescription should be restricted only to severe and progressive forms of periodontal disease to avoid the possible development of bacterial resistance [45,54]. Additionally, the Council for Appropriate and Rational Antibiotic Therapy introduced criteria to help in selecting the appropriate antibiotics, including cost-effectiveness, safety, balancing benefits/harms, optimal duration/dose, and results from the latest evidence-based studies [58].

## 4. Evidence on Using Antimicrobials/Antibiotics as Adjunct to Periodontal Therapy

### 4.1. Evidence from In Vitro and Experimental Animal Studies

Since a dysbiotic dental biofilm is the main causative agent of periodontal disease in susceptible patients, it is of paramount importance to optimize methods to identify and quantify the microbial communities within dental biofilm [59,60,61]. To achieve this, many in vitro biofilm models have been introduced, mainly to investigate antimicrobial effect of eradicating periodontal pathogens on one hand, and to support the clinical application of these drugs on the other [62,63,64]. The evolution of biofilm modeling developed from mere observation of growth and maturation on static models such as hydroxyapatite or titanium disk to more advanced dynamic systems imitating the clinical environment has allowed a much closer insight into antimicrobial action [65].

A number of studies using various antimicrobials against periodontal pathogens have been carried out (Table 1). It is apparent that the antimicrobials can kill periodontal pathogens in in vitro biofilm models. However, some studies indicated that AMX + MET were not efficient in reducing the bacterial count [66,67]. Nevertheless, most of the studies consistently reported that the combination of these two antibiotics was superior to using either of them alone, particularly against red complex bacteria [68,69,70,71]. Similar results with regard to these bacteria were obtained with other antibiotics including azithromycin (AZM) [68,71], minocycline [72], and active organic ingredients of mouthrinses [73,74,75]. However, it is important to acknowledge that owing to greater tolerance to antimicrobials, the minimum inhibitory concentration calculated in in vitro studies would purportedly be lower and would bear little relevance to in vivo situations [34,76]. Furthermore, the majority of these studies used laboratory strains in their biofilm models, which apparently differ from clinical strains in their behavior and resistance to antimicrobials [77].

The results of these in vitro studies have shown that complete eradication of bacteria did not occur, although high concentrations of these antimicrobials were used. Once more, this highlights the importance of using mechanical SD to disrupt biofilm and making limited use of these agents as adjuncts to mechanical SD, especially in deep and residual pockets [72,78,79]. Nevertheless, development of biofilm models is essential to assess growth and maturation of these microbial communities and their behavior during exposure to different antimicrobial agents, especially their sensitivity and resistance to the antimicrobial agent of interest [65].

### 4.2. Evidence from Observational Studies

Worldwide, the inappropriate use of antibiotics, together with their use non-medically, enhances bacterial resistance of microbiomes of the body, including periodontal pathogens. A study on Columbian patients with periodontitis revealed that 44% of them consumed antibiotics without prescription. Subgingival anaerobic periodontal pathogens isolated from those patients showed resistance to a range of antibiotics, particularly MET [81].

Although development of bacterial resistance of key periodontal pathogens is continually being reported, susceptibility to AMX and MET was not significantly changed [82]. In contrast, bacterial resistance of *A. actinomycetemcomitans* and different strains of *Enterococcus*. in particular was observed against these antibiotics in other studies [81,83,84,85,86,87]. In addition, subgingival bacteria exhibited varying degrees of resistance against other antibiotics such as AZM and erythromycin [81,85,87,88,89]. However, most of the pathogenic bacteria, including the red complex group, were susceptible to moxifloxacin [84,88]. Furthermore, in the last decade, many resistance genes were detected in the microbiome associated with periodontal health and disease, such as bla_CfxA_, bla_TEM_ [83], *erm* bla*TEM*, *mec*A, and *pbp*2b [90], and *tet*(32) [91]. Bacteria carrying these genes potentially transfer them to other bacterial species, leading to the development of resistance. This could explain why antibiotics sometimes fail to achieve desirable results when combined with periodontal therapy. The results from these studies have aroused skepticism about the efficiency of most of the first- and second-choice antibiotics commonly prescribed as adjuncts to periodontal therapy. Selected observational studies are summarized in Table 2 to highlight the antimicrobial resistance pattern.

The main limitations of these surveys are their dependence on isolating bacteria from subgingival domains and then testing their susceptibility to different antibiotics/antimicrobials in vitro. Although this is a validated method and can give reliable results, the behavior of these bacteria in vivo could be entirely different due to their co-existence with other microorganisms in a complex biofilm that could alter their susceptibility to antibiotics. In addition, many pathogenic bacteria cannot be grown with a conventional culturing technique due to their fastidious growth requirements. Furthermore, cross-sectional studies in this review were conducted in different countries where the bacterial-resistance pattern could greatly vary. Despite these limitations, the conclusions that could be drawn from these studies suggest that no single antibiotic is efficient against the subgingival microbiome, which is inhabited by a diverse range of pathogenic bacterial species, most of which exhibit resistance to the most commonly used antibiotics in periodontal therapy. Therefore, testing antimicrobial susceptibility is essential before prescribing any antibiotic(s). However, this is not practical in a general dental environment where the best approach to avoid developing bacterial resistance is by avoiding unnecessary prescriptions.

### 4.3. Evidence from Clinical Trials

Many studies have been conducted to evaluate the additional benefits of using antibiotics within the course of periodontal therapy. Results from some of these studies have concluded that antibiotics are important adjuncts to SD in specific situations [93,94,95]. The additional clinical benefits of antibiotics are more pronounced in molar sites than in non-molar sites [42]. Following SD, the administration of broad-spectrum antibiotics for three to seven days improves microbiological outcomes compared to SD alone [95]. In regenerative periodontal therapy, better clinical outcomes could be achieved when systemic antibiotics are prescribed for patients [96].

On the other hand, many studies have reported that the use of antibiotics as an adjunct to periodontal therapy has no additional clinical benefits. Following periodontal surgery, the adjunctive use of AMX alone [97] or in combination with MET [98] for more than one week postoperatively provides no additional clinical improvements after one year. Similarly, the use of AZM as an adjunct to SD for the treatment of periodontitis seems to have no role in improving clinical outcomes compared to SD alone [99] despite the reduction in the levels of periodontal pathogens in deep periodontal pockets [100].

In type 2 diabetic patients, the use of antibiotics as an adjunct to SD is still under debate, and no conclusive evidence can be drawn. It was reported that the adjunctive use of antibiotics after SD adds no clinical benefits [101]. In contrast, the use of post-operative antibiotics has been found to induce clinical benefits for up to two years for such patients [102].

The use of local antibiotics as adjuncts to SD for treating deep periodontal pockets has been evaluated in many studies. Compared to SD alone, better clinical outcomes could be achieved when antibiotics are locally applied, as gel or microsphere, into pockets after SD [103,104]. However, no clinical benefit of using local antibiotics as adjuncts to SD was reported [105].

In conclusion, the clinical benefits of using antibiotics as an adjunct to periodontal therapy are not clear in the literature. Reviewing the literature has revealed conflicting evidence in the outcomes of clinical studies (Table 3). In order to better understand any possible efficacy of antibiotic use on clinical outcomes, more well-controlled clinical trials are needed.

## 5. Novel Antibacterial Agents and Strategies to Overcome Bacterial Resistance in Dental Biofilm: Pros and Cons

Interest in seeking novel alternative adjuncts to SD was raised due to limitations of conventional SD methods [108,109] and drawbacks of antimicrobials/antibiotics. Antimicrobial photodynamic therapy (aPDT) and laser are among the suggested methods that have been thoroughly investigated. Additionally, probiotics emerged as another promising approach to prevent and treat periodontal disease [110,111].

The use of lasers to debride periodontal pockets and ablate subgingival deposits has gained some traction in dental practice [112]. The utilization of a low-level laser light in combination with a photosensitizer, e.g., toluidine blue, is known as aPDT. The principle of this technique is based on light exposure of a photosensitizer releasing highly reactive oxygen radicals which destroy bacteria in periodontal pockets [113]. Additionally, photonic energy is presumed to enhance tissue healing by bio-stimulatory effects; further improvement of clinical parameters is therefore expected, such as the reduction of PPD and BOP, as well as CAL gain [114]. Concomitant improvement in clinical and microbiological parameters when aPDT was used as adjunct to SD was reported in several studies [115,116,117,118]. This was consistent with the results from a current systematic review and meta-analysis that have shown positive effects on the clinical outcomes of using aPDT together with laser, with a high impact on key periodontal pathogens, particularly the red complex [119]. However, other trials showed only a reduction of periodontal pathogens without significant difference in clinical parameters when compared to SD only [120,121]. In addition, results from other studies indicated that neither microbiological nor clinical parameters were improved following the application of laser or aPDT [122,123,124,125,126,127,128] (Table 4). Overall, the results of the current review were consistent with guidelines issued by the European Federation of Periodontology (EFP), which suggested that there is no clear evidence supporting the use of lasers or PDT as adjuncts to SD [29].

The concept of probiotics was first suggested by a Russian scientist, Elie Metchnikoff, in the early 20th century [129]. Probiotics can be defined as live/inactivated microorganisms or their components that when administered in certain amounts exert beneficial health effects on the host [130,131]. The principle of probiotic action relies on two mechanisms: the elimination of certain pathogens and modulating an aberrant host’s immune responses [110]. Although use of probiotics for improving general health dates back to the 1950s, the application of probiotics to the oral cavity is a relatively new concept [132]. Within the scope of the current review, certain studies were selected to demonstrate the clinical/microbiological effects of using probiotics as adjuncts to SD (Table 5). Only two studies showed positive effects on both parameters when genera of *Bifidobacterium* (1 × 10^9^ CFU) were administrated twice daily over 30 days [133,134]. However, the majority of included studies involving *Lactobacillus* and other species showed improvement either in clinical or microbiological parameters [135,136,137,138]. The lack of any significance difference in clinical and microbiological outcomes at the end of the trial when compared to control was observed in other studies [139,140]. In fact, results from the work of Pudgar and coauthors (2021) showed that higher numbers of residual pockets of 4 mm with BOP was associated with the probiotic group when compared to the control arm [139]. The findings of this review aligned with the current guidelines issued by the EFP that suggests not administrating probiotics as an adjunct to SD due to the heterogeneity of trials and lack of conclusive evidence supporting their use [29].

Bacteriophage therapy is another suggested approach to regaining the symbiotic state of periodontal microbiota. Briefly, this concept relies on administrating a virus (phage) which in turn infects and subsequently controls the population of a specific pathogenic bacterium [141]. Although phage therapy is characterized by specificity and selectivity, it requires knowledge about the targeted bacterium with the possibility of developing resistance [142]. Besides, the application of bacteriophages in periodontal therapy is still limited to in vitro studies [143]. Nevertheless, utilizing the predator-prey relationship, i.e., bacteriophages vs. periodontal pathogens in treating periodontal disease, is appealing and could be a successful way to overcome increasing bacterial resistance against the available antibiotic arsenal. No clinical trials have yet been conducted to confirm or deny the validity of this concept in periodontal therapy, and it remains an experimental approach until fully investigated.

The use of medicinal plants is another attractive alternative due to the safety of using natural herbal-based products. Many studies have been conducted to investigate the beneficial effects of using medicinal plants for plaque control and prevention of periodontal diseases. For instance, the *Salvadora persica L.* chewing stick as a natural toothbrush could be used as a potent alternative to artificial toothbrushes for plaque control with better anti-gingivitis effects [144]. As a mouthwash, *Salvadora persica L.* extracts exert antiplaque effect and significantly reduce cariogenic bacterial counts i.e., *Streptococcus mutans* and *Lactobacillus counts* [145]. There is an evidence that oral preparations of *Camellia sinensis* leaves extract potentially help in controlling dental biofilm and adjunct the management of gingivitis [146]. Toothpastes containing herbal extracts such as *Aloe vera*, *chamomile*, *Salvadora persica* and *chitosan* do not enhance more reduction of dental biofilm than non-herbal toothpastes in long-term use. On the other hand, herbal mouthwashes as antiplaque agents are inferior to the gold standard chlorhexidine mouth wash [147]. According to a recent systematic review and meta-analysis, there is low to moderate evidence that natural antimicrobials derived from phenolic compounds are as effective as chlorhexidine in reducing oral microorganisms counts but not for controlling dental biofilm [148]. However, the available clinical trials exhibit a high degree of heterogeneity and risk of biases that limit the justification of using these products [149,150]. More attention should be given to the methodological protocols of clinical trials investigating antiplaque and antimicrobial effectiveness of variety of plants extracts. In particular, it is important to standardize the dosage and duration of plant extracts interventions to obtain comparable results among studies and achieve solid conclusions [146].

In summary, a high degree of heterogeneity can be recognized in the selected studies regarding laser and aPDT such as wavelengths, single vs. multiple applications, light dose and type of photosensitizer. In addition, a carry-over effect could compromise the results from studies which followed a split-mouth design. Furthermore, applying a photosensitizer within periodontal pockets could disturb and remove the biofilm mechanically by a flushing effect. Similarly, studies on probiotics also showed heterogeneity, mainly through using different probiotic strains which were administrated in various modes, dosage, and frequency. Generally, the majority of trials on aPDT and probiotics in combination with SD have evaluated their efficacy only in the short term, i.e., three-months, and few studies extended up to 12-months. Additionally, these studies exhibited differences in the microbiological techniques and bacterial species investigated. Despite promising results being observed in some trials, the level of evidence supporting the use of these modalities as adjuncts to periodontal treatment is still low. Therefore, further well-designed, controlled, randomized clinical trials with a longer follow-up period are required to draw confirmatory conclusions.

## 6. Conclusions

Antibiotics/antimicrobials cannot be used as a monotherapy for treating periodontal disease and must be combined with mechanical debridement for biofilm disruption. Wherever possible, antibiotic use should be limited and appropriate to reduce the risk of adding to bacterial resistance. In particular, antibiotics should not be used in the vast majority of cases of periodontitis and only in specific cases such as young adults with rapidly progressing periodontitis i.e., stage III/IV, grade C and when the associated risk factors are inconsistent with the amount of bone loss present. In addition, the prescription of antibiotics is recommended following an intense and short course of SD.

According to RCTs, the recommended antibiotic regimen to prescribe as an adjunct to SD is a combination of AMX + MET (500/400 mg) for 7 days. However, these antibiotics should be prescribed with caution, as they are associated with the highest adverse effects and bacterial resistance.

Overall, long-term efficacy of other alternative therapies, e.g., laser/aPDT and probiotics, is not well-reported in the literature and the results do not currently support their use as adjuncts to SD.

## Figures and Tables

**Table 1:** Summary of in vitro studies on efficacy of commonly used antimicrobial agents against periodontitis-associated bacteria.

Antimicrobial	Bacterial Species *	Outcome	Publications
AMX, MET, or their combination	*Ag, Ai, Ao, Aod, Vp, Sg, Si, Sm, So, Ss, Sa, Smu, Aa, Cg, Co, Cs, Ec, Cc, Cgr, Cr, Csh, En, Es, Fn, Fnp, Fnv, Fp, Pm, Pi, Pn, Pme, Sn, Tf, Pg, Gm, Lb, Nm, Td, Pa*, and *Sno*	Combination of AMX and MET exhibited greater antimicrobial effects than using each antibiotic seperately.	[68,69,70,71]
	*Ss, Fn,* and *Pg*	Growth rate was reduced in response to either AMX or MET but not their combination.	[66]
	*To, Sa, Ao, Fn, Vd, Cr, Pi, Pg, Tf* and *Td*	Antibiotics caused species-specific reductions, but not total bacterial loads	[67]
AZM	*Pg, Td, Tf,*	AZM was ineffective in preventing biofilm formation within a clinically achievable concentration.	[68,71]
	*So, Sa, Ao, Fn, Vd, Cr, Pi, Pg, Tf,* and *Td.*	Total bacterial counts were significantly reduced	[67]
MNO	*Pg, Fn, Tf, Sg, An,* and *Pm*	The antimicrobial activity of MNO reduced total cfu of examined species.	[72]
DOX	*Pg* and *Fn*	Substantial antimicrobial activity of DOX against periodontal pathogens.	[80]
CHX and CPC	*An, Ao, Ag, Ai, Vp, Aod, Ss, So, Si, Sg, Sm, Aa, Co, Cg, Ec, Cs, Sc, En, Fnv, Pm, Fnp, Csh, Fn, Fp, Pi, Pg, Tf, Es, Sa, Sno, Pa,* and *Gm.*	CHX/CPC demonstrated superior antimicrobial activity. CHX specifically reduced levels of *Fnv* and *Pg* while CPC was more effective against *Aod* and *Ai*.	[73,74,75]

* Bacteria examined are all laboratory strains. *Ag; Actinomyces gerencseriae, Ai; Actinomyces israelii, An; Actinomyces naeslundii, Ao; Actinomyces oris, Aod; Actinomyces odontolyticus, Vp; Veillonella parvula, Sg; Streptococcus gordonii, Si; Streptococcus intermedius, Sm; Streptococcus mitis, So; Streptococcus oralis, Ss; Streptococcus sanguinis, Sa; Streptococcus anginosus, Smu; Streptococcus mutans, Aa; Aggregatibacter actinomycetemcomitans, Cg; Capnocytophaga gingivalis, Co; Capnocytophaga ochracea, Cs; Capnocytophaga sputigena, Ec; Eikenella corrodens, Cc; Campylobacter concisus, Cgr; Campylobacter gracilis, Cr; Campylobacter rectus, Csh; Campylobacter showae ATCC 51146, En; Eubacterium nodatum, Es; Eubacterium saburreum, Fn; Fusobacterium nucleatum subsp. nucleatum, Fnp; Fusobacterium nucleatum subsp. polymorphum, Fnv; Fusobacterium nucleatum subsp. vincentii, Fp; Fusobacterium periodonticum ATCC 33693, Pm; Parvimonas micra, Pi; Prevotella intermedia, Pn; Prevotella nigrescens, Pme; Prevotella melaninogenica, Sn; Streptococcus constellatus, Tf; Tannerella forsythia, Porphyromonas gingivalis, Gm; Gemella morbillorum, Lb; Leptotrichia buccalis, Nm; Neisseria mucosa, Pa; Propionibacterium acnes, Sno; Selenomonas noxia, Sc; Streptococcus constellatus. To; Treptococcus oralis, Vd; Veillonella dispar, Td; Treponema denticola.* AMX; Amoxicillin, MET; Metronidazole, AZM; Azithromycin, CHX; Chlorhexidine, DOX; doxycycline, PV; penicillin V, CPC; Cetylpyridinium chloride, MNO; Minocycline, GCF; gingival crevicular fluid, cfu; colony forming unit.

**Table 2:** Summary of observational studies for resistance pattern of subgingival biofilm bacteria against common antibiotics in periodontal health and disease.

Antibiotic	Resistant Bacteria	Publications
Amoxicillin	*Rd, Fn, Tf, Aa, Pg, Pi, Streptococcus* spp., *Enterococci* spp.	[81,82,83,84,85,86,88,92]
Metronidazole	*Rd, Ga, An, Aa, Pg, Tf, Pi, Fn*	[81,84,86,88,92]
Penicillin	*An, Aa*	[86,92]
Amoxicillin/clavulanic acid	*Rd, Fn, Aa*	[82,86,92]
Azithromycin	*Ec, An, Pg, Pi, Fn, Aa, Tf*	[82,84,89,92]
Tetracyclin	*An, Aa, Ef*	[86,87,92]
Erythromycin	*Pi, Streptococcus* spp., *EF,*	[83,85,87,89]
Ciprofloxacin	*Enterococci* spp.	[85]
Clindamycin	*Enterococci* spp., *Aa, Pg*	[85,86,88]

*Rd: Rothia dentocariosa, An: Actinomyces naeslundii, Ga: Granulicatella adiacens, Ec: Eikenella corrodens, Ef: Enterococcus faecalis, Fn: Fusobacterium nucleatum, Aa: Aggregatibacter actinomycetemcomitans, Pg: Porphyromonas gingivalis, Tf: Tannerella forsythia, Pi: Prevotella intermedia.*

**Table 3:** Summary of randomized clinical trials on efficacy of antibiotics as adjunct to nonsurgical periodontal therapy.

Author, Year	Type of Treatment	Sample (n)	Antibiotic Dose/Frequency	Follow-Up	Periodontal Parameters
No improvement in clinical parameters					
Morales et al., 2021 [99]	SD for stage III periodontitis patients	control: SD (n = 15); test: SD+ probiotics (n = 16) test: SD + AB (n = 16)	500 mg of AZM 1/day for 5 days	12-months	PI, BOP, PPD, and CAL
Qureshi et al., 2021 [101]	SD and OHI for T2DM patients with periodontitis	control: OHI (n = 50) control: SD + OHI (n = 50) test: AB + SD + OHI (n = 50)	400 mg of MET 3/day for 10 days	3- and 6-months	BOP, PPD and CAL
Serino et al., 2001 [106]	SD for patients with recurrent advanced periodontitis	17 received SD + AB	400 mg of MET 3/day + 750 mg AMX 2/day for 2 weeks	1, 3, 5 years	PI, BOP, PPD, PAL and radiographic bone level
Choi et al., 2021 [105]	SD periodontitis patients	control: SD (n = 12) test: SD + 2% minocycline	microcapsule gel containing 2% minocycline HCl ointment	1- and 3-months	PI, BOP, PPD, CAL and relative ratios of periodontal pathogens
Harks et al., 2015 [93]	SD + maintenance therapy at 3 months intervals.	control: SD (n = 200) test: SD+AB (n = 206)	500 mg AMX + 400 mg MET 3/day for 7 days	27.5-months	percentage of sites showing further attachment loss, measurements from occlusal surface to the pocket bottom
Improvement in clinical parameters only					
Cosgarea et al., 2020 [95]	SD for severe periodontitis patients	control: (n = 26) test: AMX + MET for first 3 days: (n = 24); AMX+MET for 7 days: (n = 25)	500 mg of AMX thrice a day 500 mg of MET 3/day	3-, 6- and 12-months	PI, BOP, PPD, CAL and number of deep sites with PPD ≥ 6 mm,
Mombelli et al., 2013 [42]	full-mouth SD within 48 hrs for moderate to advanced periodontitis patients	control: only SD (n = 38) test: SD+AB (n = 44)	375 mg of AMX + 500 mg of MET, 3/day for 7 days	3-months	Persistence of sites with a probing depth (PD) >4 mm and BOP
Trajano et al., 2020 [103]	SD	control: SD test: 10% doxycycline in β-cyclodextrin +SD test: 10% doxycycline +SD	gel of 10% doxycycline in β-cyclodextrin or alone applied at baseline and after a month	30 and 60 days	PI, BOP, PPD, and CAL
Mombelli et al., 2005 [96]	SD + enamel matrix derivatives for periodontitis patients	control: SD (n = 8) test: AB+SD (n = 8)	375 mg of AMX + 250 mg of MET 3/day for 7 days	6- and 12-months	PPD and CAL
Cruz et al., 2021 [102]	SD for T2DM patients with periodontitis. None had received SD from 2 to 5 years post-treatment	control: SD (n = 10) test: SD+AB (n = 15)	400 gm MET+500 mg AMX 3/day for 14 days and started at the first SD session	up to 5 years	PI, BOP, PPD, CAL and number of sites with PD ≥ 5 mm
Ali et al., 2021 [104]	SD for mild to moderate periodontitis patients	control: SD (n = 24) test: SD+ Lycopene (n = 24) test: SD + minocycline HCL (n = 24)	minocycline HCL microspheres and lycopene gel	30 days	PI, BOP, PPD and CAL
Improvement in microbilogical parameters only					
Cosgarea et al., 2021 [107]	SD for periodontitis patients	control: SD (n = 35) test: SD + LDD (n = 35)	LDD	3- and 6-months	PI, BOP, PPD, CAL, number of treated sites with BOP and 8 periodontopathogens levels
Čuk et al., 2020 [100]	SD for periodontitis patients	control: SD (n = 20) test: SD + AB (n = 20)	AZM 500 mg/day for 3 days	6-months	N of sites with PD ≥ 5 mm and BOP, changes in numbers of periodontal pathogens in pockets

AB: antibiotic; AZM: azithromycin; MET: metronidazole; AMX: amoxicillin; LDD: locally delivered doxycycline; SD: subgingival debridement; CAL: clinical attachment level; PPD: probing pocket depth; BOP: bleeding on probing; PI: plaque index; OHI: oral hygiene instructions; PAL: probing attachment level; PBL: probing bone level; GR: gingival recession; T2DM: type 2 diabetes mellitus.

**Table 4:** Efficacy of laser and antimicrobial photodynamic therapy as adjuncts to nonsurgical periodontal therapy on microbiological and clinical parameters.

Author, Year	Study Design, Follow-Up	Study Population	Clinical/Microbiological Parameters	aPDT Treatment Modalities
Improvement in microbiological and clinical parameters ^§^				
Moreira et al., 2015 [118]	Split-mouth RCT, 3-months	Patients with generalized AgP (n = 20)	PI, BOP, PPD, REC, CAL 40 bacterial species using the checkerboard DNA–DNA hybridization technique	SD + Diode laser (670 nm)/phenothiazine chloride (10 mg/mL) photosensitizer
Gandhi et al., 2019 [116]	Split-mouth, RCT, 9-months	Periodontitis patients (n = 26)	PPD, PI, GI, CAL Count of *Pg, Aa*	SD + Diode laser (810 nm)/ICG photosensitizer
Annaji et al., 2016 [117]	Split-mouth RCT, 3-months	Patients with AgP (n = 15)	PI, BOP, RAL, PPD Culture method to identify *Pg, Aa, Pi*	SD+ Diode Laser (810 nm)
Wadhwa et al., 2021 [115]	Split-mouth RCT, 6-months	Chronic periodontitis patients (n = 30)	Total viable anaerobic count	SD + Diode laser (810 nm)/ICG photosensitizer
Improvement in microbiological parameters only ^§^				
Muzaheed et al., 2020 [120]	Parallel arm RCT, 3-months	Periodontitis patients (n = 45)	PI, CAL, PPD, GI Culture method to identify *Pg*, *Aa*, *Td*, *Pi*, *Fn*	SD + Diode laser (660 nm)/methylene-blue (0.005%) photosensitizer
Chondros et al., 2009 [121]	Parallel arm RCT, 6-months	Periodontitis patients (n = 24)	PPD, REC, CAL, FMPS, FMBS Quantification of *Pg*, *Aa*, *Td*, *Pi*, *Tf, Fn, Pm, Cr, En, Ec, Cs* by PCR	SD + Diode Laser (670 nm)/phenothiazine chloride (10 mg/mL) photosensitizer
No improvement in microbiological and clinical parameters ^§^				
Chitsazi et al., 2014 [127]	Split-mouth RCT, 3-months	Patients with AgP (n = 24)	PPD, CAL, REC, BOP, PI, GI Quantification of *Aa* by PCR	SD + Diode Laser (670–690 nm)
Rühling et al., 2010 [128]	Parallel arm RCT, 3-months	Periodontitis patients (n = 54)	PI, PPD, CAL, BOP Quantification of *Pg*, *Aa*, *Td*, *Pi*, *Tf, Fn* by PCR	SD + Diode Laser (635 nm)/5% tolonium chloride photosensitizer
Queiroz et al., 2015 [125] Queiroz et al., 2014 [126]	Parallel arm RCT, 3-months	Periodontitis smoker patients (n = 20)	PI, BOP, PPD, CAL, REC 40 bacterial species using the checkerboard DNA–DNA hybridization technique	SD + Diode Laser (660 nm)/phenothiazine chloride (10 mg/mL) photosensitizer
Tabenski et al., 2017 [123]	Parallel arm RCT, 12-months	Periodontitis patients (n = 45)	API, PBI, BOP, PPD, CAL molecular-biological testing system to identify *Pg*, *Aa*, *Td*, *Tf* + TML and TBL	SD + Diode Laser (670 nm)/phenothiazine chloride photosensitizer
Hill et al., 2019 [122]	Split-mouth RCT, 6-months	Periodontitis patients (n = 20)	BOP, PPD, RAL, REC Quantification of *Pg*, *Aa*, *Td*, *Pi*, *Tf* by PCR	SD + Diode laser (808 nm)/ICG photosensitizer
Pulikkotil et al., 2016 [124]	Split-mouth RCT, 3-months	Periodontitis patients (n = 20)	BOP, PPD, CAL Quantification of *Aa* by PCR	SD + LED lamp (red spectrum, 628 Hz)/methylene blue photosensitizer

NSPT: nonsurgical periodontal therapy, aPDT: antimicrobial photodynamic therapy, RCT: randomized clinical trial, AgP: aggressive periodontitis, SD: subgingival debridement, PI: plaque index, PPD: probing pocket depth, CAL: clinical attachment level, RAL: relative attachment level, BOP: bleeding on probing, GI: gingival index, REC: recession, FMPS: full-mouth plaque score, FMBS: full-mouth bleeding score, PCR: polymerase chain reaction, ICG: indocyanine green, *Pg*: *Porphyromonas gingivalis*, *Aa: Aggregatibacter actinomycetemcomitans*, *Td: Treponema denticola*, *Pi*: *Prevotella intermedia*, *Fn*: *Fusobacterium nucleatum, Tf: Tannerella forsythensis, Pm: Peptostreptococcus micros, Cr: Campylobacter rectus, En: Eubacterium nodatum, Ec: Eikenella corrodens, Cs: Capnocytophaga species*, TML: total marker load, TBL: total bacterial load, API: approximal plaque index, PBI: papillary bleeding index. ^§^ Outcomes of PDT at endpoint as compared to control arm.

**Table 5:** Efficacy of probiotics as an adjunct to nonsurgical periodontal therapy on microbiological and clinical parameters.

Author, Year	Study Design, Follow-Up	Study Population	Strain of Probiotic	Mode/Frequency of Administration	Clinical/Microbiological Parameters
Improvement in microbiological and clinical parameters ^§^					
Invernici et al., 2018 [134]	Parallel arm RCT, 3-months	Chronic periodontitis patients (n = 41)	*Bl* (HN019) 1 × 10^9^ CFU	Lozenges (10 mg) 2×/day for 30-days	PI, BOP, PPD, CAL, REC 40 subgingival bacterial species were identified using the checkerboard DNA-DNA hybridization technique
Invernici et al., 2020 [133]	Parallel arm RCT, 3-months	Chronic periodontitis patients (n = 30)	*Bl* (HN019) 1 × 10^9^ CFU	Lozenges 2×/day in the morning and before bedtime for 30-days	PI, BOMP In vitro assay for adhesion of *Bl* and *Pg* to BEC Antimicrobial activity of *Bl* against *Fn, Pg, Pi,* and *Aa*
Improvement in clinical parameters only ^§^					
Laleman et al., 2020 [135]	Parallel arm RCT, 6-months	Chronic periodontitis patients (n = 39)	*Lr* (DSM 17,938 and ATCC PTA 5289) 2 × 10^8^ CFU each	Five probiotic drops applied to residual pocket immediately after SD. Then each patient instructed to use lozenges 2×/day after brushing for 3-months	PPD, REC, CAL, FMPS, FMBS PCR was used to quantify *Pg, Pi, Fn, Aa*
Tekce et al., 2015 [137]	Parallel arm RCT, 12-months	Chronic periodontitis patients (n = 30)	*Lr* (DSM 17,938 and ATCC PTA 5289) 2 × 10^8^ CFU each	Lozenges 2×/day after brushing for 3-weeks	PI, GI, BOP, PPD, CAL Total viable cell count and the proportions of obligate anaerobic bacteria were determined
Improvement in microbiological parameters only ^§^					
Dhaliwal et al., 2017 [136]	Parallel arm RCT, 3-months	Chronic periodontitis patients (n = 30)	*Sf* (T-110 JPC), 30 × 10^7^ CFU, *Cb* (TO-A HIS), 2 × 10^6^ CFU, *Bm* (TO-A JPC), 1 × 10^6^ CFU and *Ls* (HIS), 5 × 10^7^ CFU	Bifilac lozenges 2×/day or 21-days	PI, GI, PPD, CAL Microbiologic count of *Aa, Pg, Pi*
Teughels et al., 2013 [138]	Parallel arm RCT, 3-months	Chronic periodontitis patients (n = 30)	*Lr* (DSM17938 and ATCC PTA5289) 9 × 10^8^ CFU each	Lozenges 2×/day for 3-months	PPD, BOP, REC, GI, PI PCR was used to quantify *Tf, Pg, Aa, Fn, Pi*
No improvement in microbiological and clinical parameters ^§^					
Pudgar et al., 2021 [139]	Parallel arm RCT, 3-months	Chronic periodontitis patients (n = 40)	*Lb* (CECT7480) and *Lp* (CECT7481), 6.0 × 10^9^ CFU/mL each	One lozenge/day	GBI, PI, PPD, CAL, BOP, REC Culture method and MALDI TOF MS for *Pi, Pm, Fn, Ec, Cr, Ca, Pg, Tf, Aa*
Morales et al., 2018 [140]	Parallel arm RCT, 9-months	Chronic periodontitis patients (n = 47)	*Lrh* (SP1) 2 × 10^7^ CFU	One sachet in water (150 mL) and ingest it once a day after brushing for 3-months	PPD, PI, BOP, CAL Culture method and PCR to cultivate and identify *Pg, Tf, Aa,*

RCT: randomized clinical trial, *Lr*: *Lactobacillus reuteri*, PPD: probing pocket depth, CAL: clinical attachment level, BOP: bleeding on probing, *Lb: Lactobacillus brevis, Lp: Lactobacillus plantarum*, GBI: gingival bleeding index, PI: plaque index, REC: recession, FI: furcation involvement, MALDI TOF MS: matrix assisted laser desorption/ionization time-of-flight mass spectrometry, *Pi: Prevotella intermedia, Pm: Parvimonas micra, Fn: Fusobacterium nucleatum, Ec: Eikenella corrodens, Cr: Campylobacter rectus, Ca: Capnocytophaga ochracea, Pg: Porphyromonas gingivalis, Tf: Tannerella forsythia*, GI: gingival index, *Lr: Lactobacillus Rhamnosus*, PCR: polymerase chain reaction, *Ba: Bifidobacterium animalis*, FMPS: full-mouth plaque scores, FMBS: full-mouth bleeding scores, *So: Streptococcus oralis*, *Su: Streptococcus uberis*, *Sr: Streptococcus rattus*, *Bl: Bifidobacterium animalis subsp. Lactis*, BOMP: bleeding on marginal probing, BEC: buccal epithelial cells, *Sf: Streptococcus faecalis, Cb: Clostridium butyricum, Bm: Bacillus mesentericus, Ls: Lactobacillus sporogenes.* ^§^ Outcomes of probiotic treatment at endpoint as compared to control arm.

## Data Availability

The data presented in this study are available on request from the corresponding author.
